# Supplementary material for: Maize transposable elements contribute to long non-coding RNAs that are regulatory hubs for abiotic stress response
Source: BMC Genomics. 2019 Nov 15;20:864. doi: 10.1186/s12864-019-6245-5 (PMC6858665; doi:10.1186/s12864-019-6245-5)

Supplemental Figures:

Figure S1: Bioinformatics pipeline for lncRNA identification, content and response to abiotic stress.

Figure S2: Chromosome distribution of long non-coding RNAs. Density were plotted on each chromosome.

Figure S3: Differential expressed genes under different abiotic stresses. A-D, Venn diagram of DEGs under stress condition across different development stage V3-V6. E-G, Venn diagram of DEGs under different stress conditions at one stage.

Figure S4: the relationships between module and trait.

Figure S5-9: the specific expression profiles of eigen-lncRNAs under different abiotic stresses across developmental stages.

Figure S1: Bioinformatics pipeline for lncRNA identification, content and response to abiotic stress.

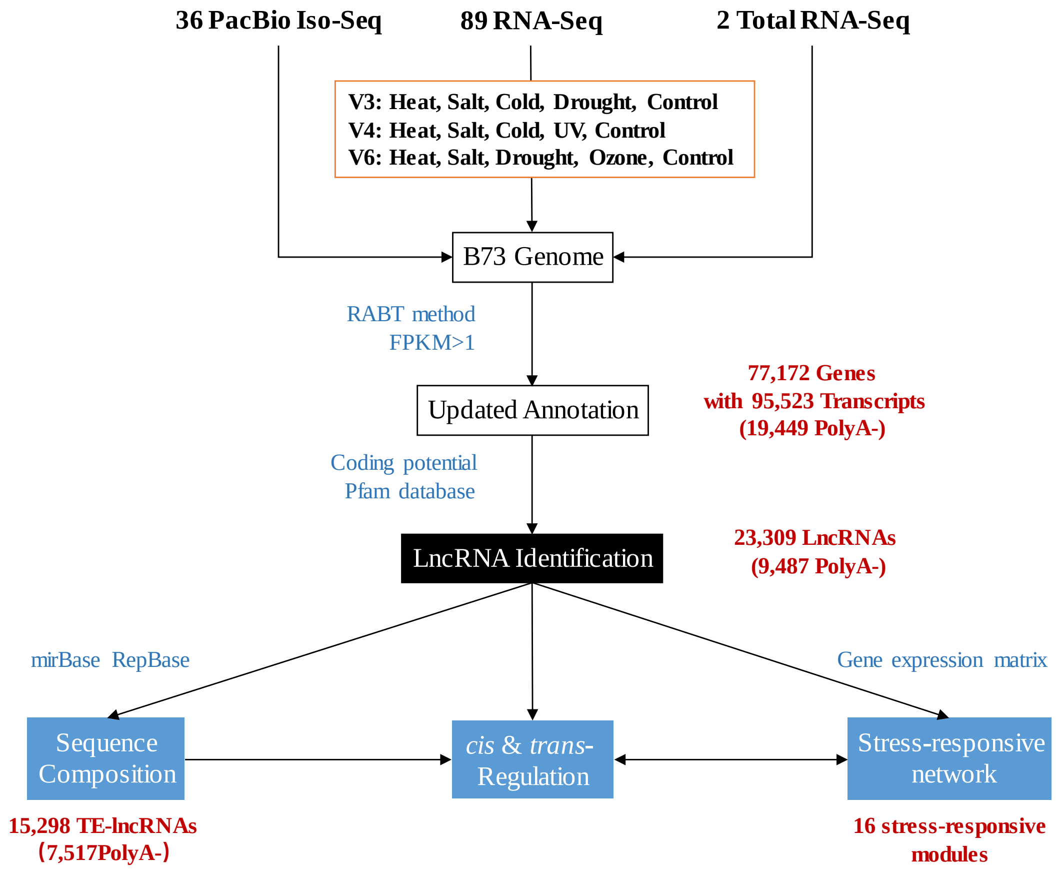

Figure S2: Chromosome distribution of long non-coding RNAs. Density were plotted on each chromosome.

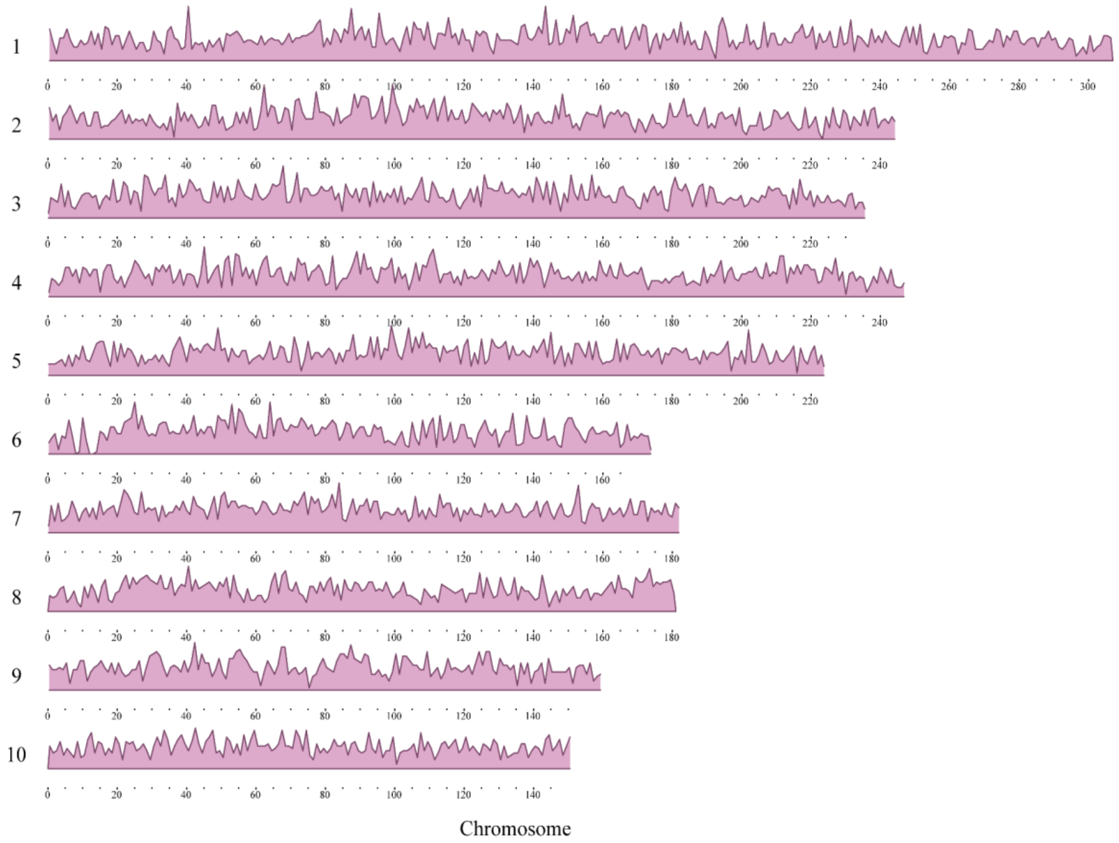

Figure S3: DE-lncRNAs under abiotic stress

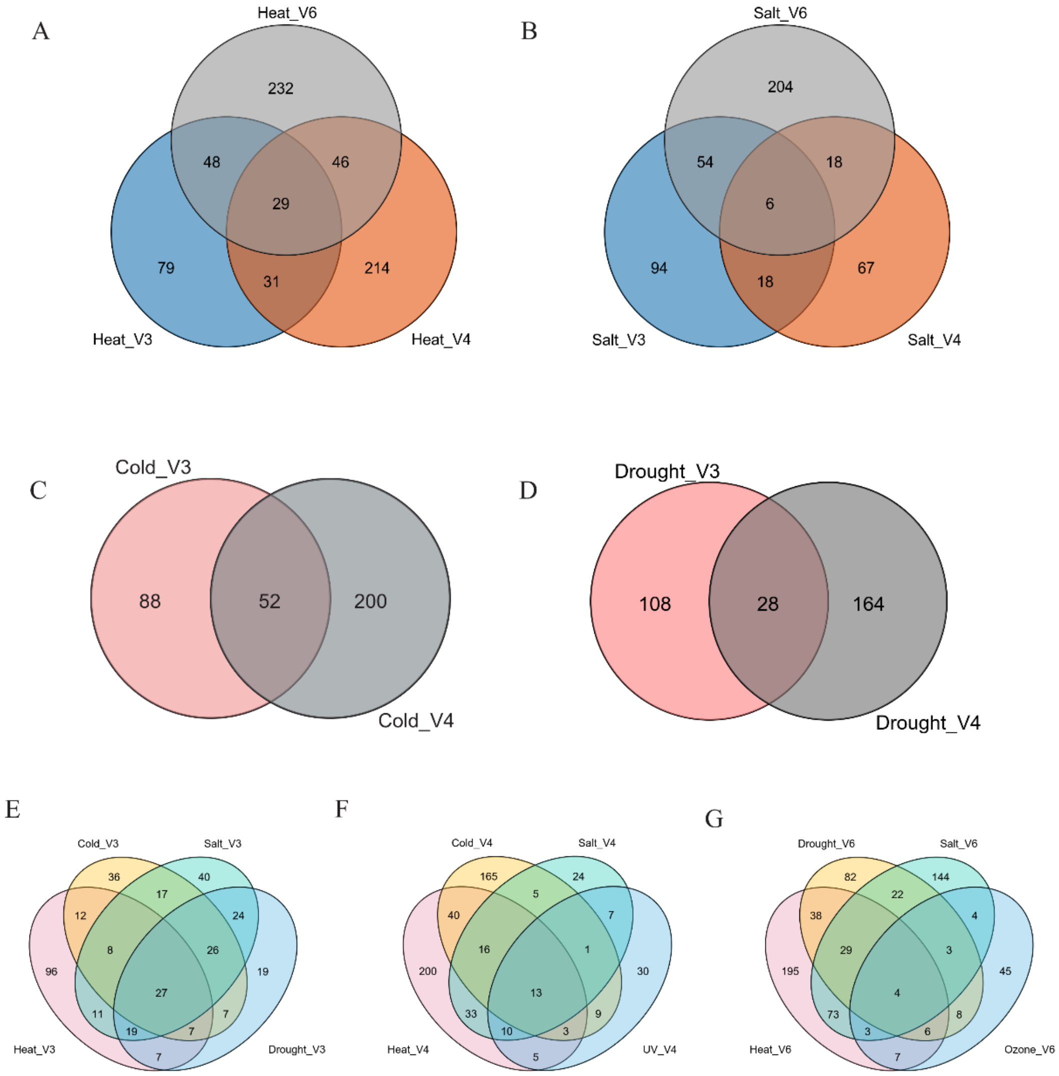

Figure S4: the relationships between module and trait.

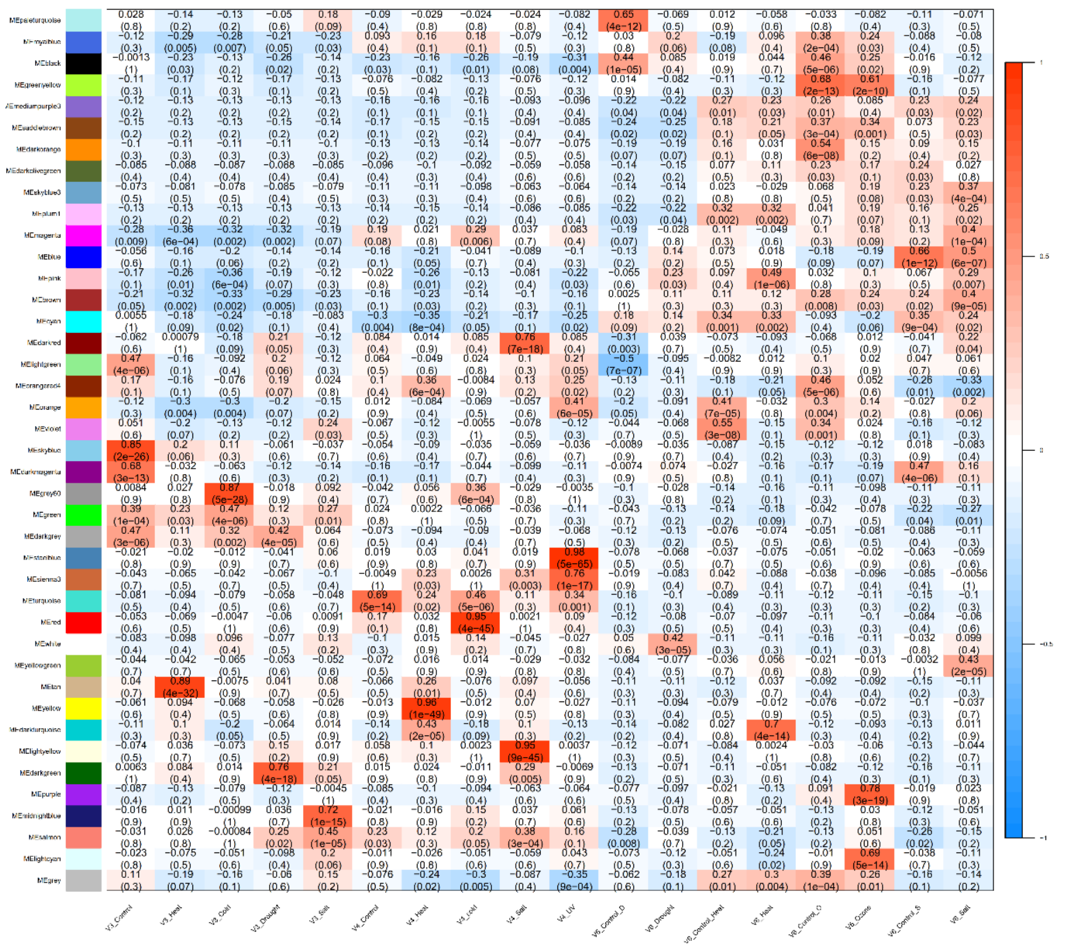

Figure S5: The expression profile of hub genes in cold-associated modules

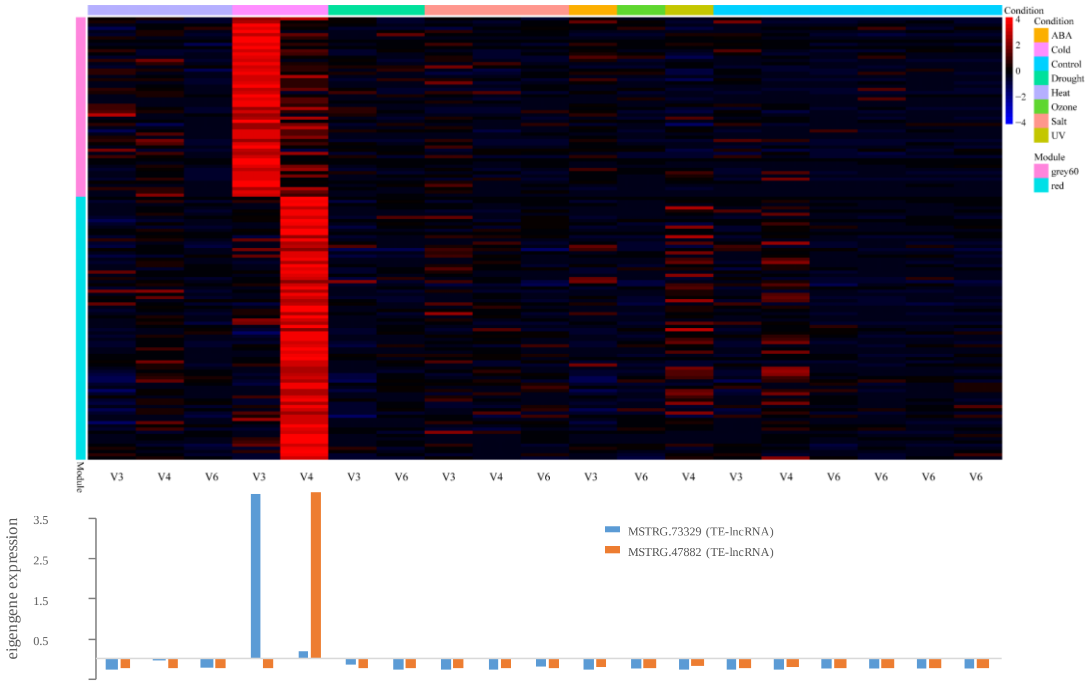

Figure S6: The expression profile of hub genes in drought-associated modules

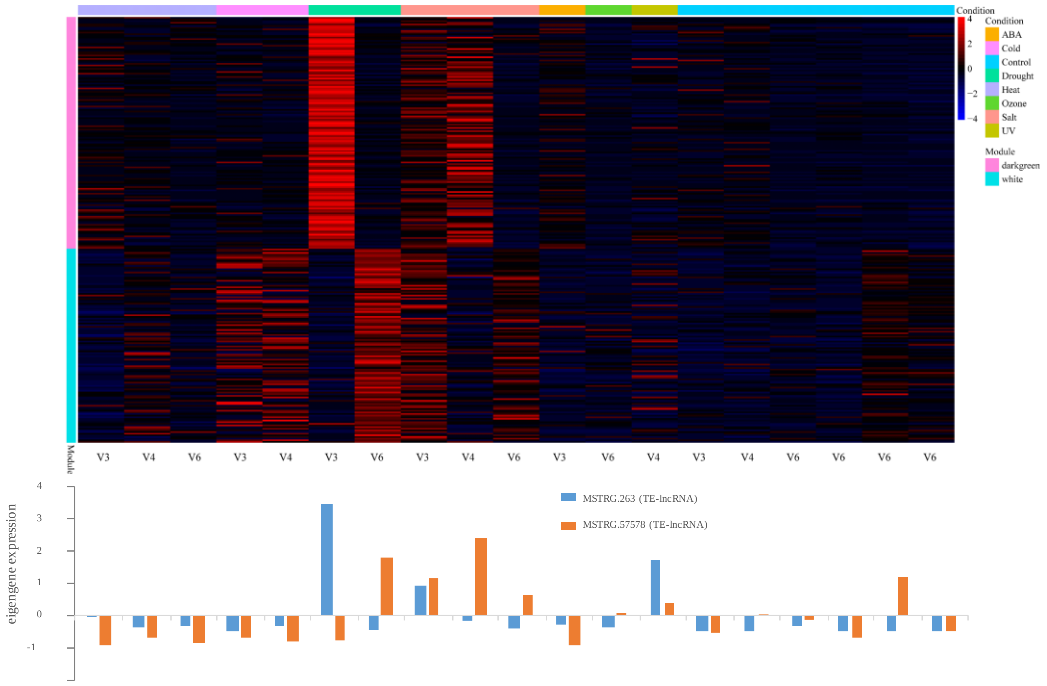

Figure S7: The expression profile of hub genes in salt-associated modules

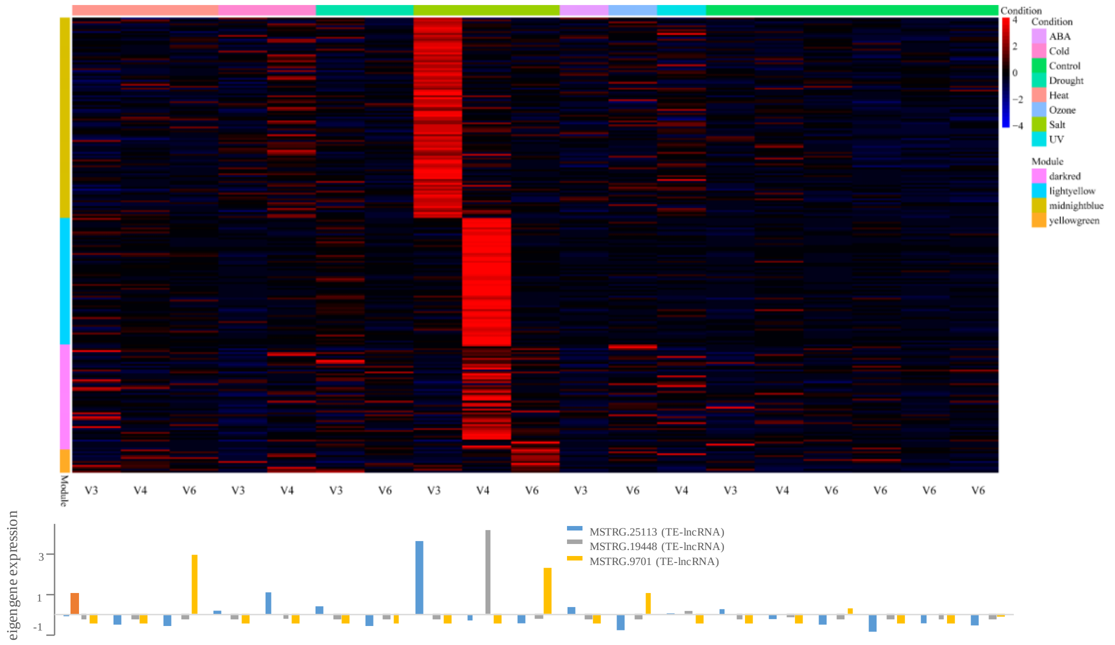

Figure S8: The expression profile of hub genes in UV-associated modules

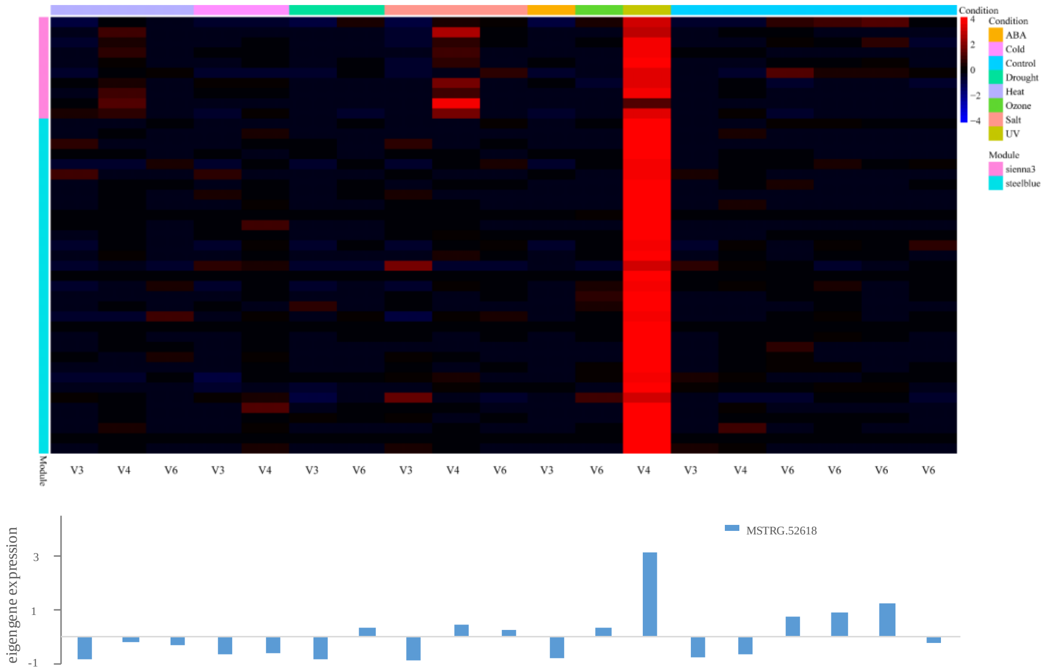

Figure S9: The expression profile of hub genes in ozone-associated modules

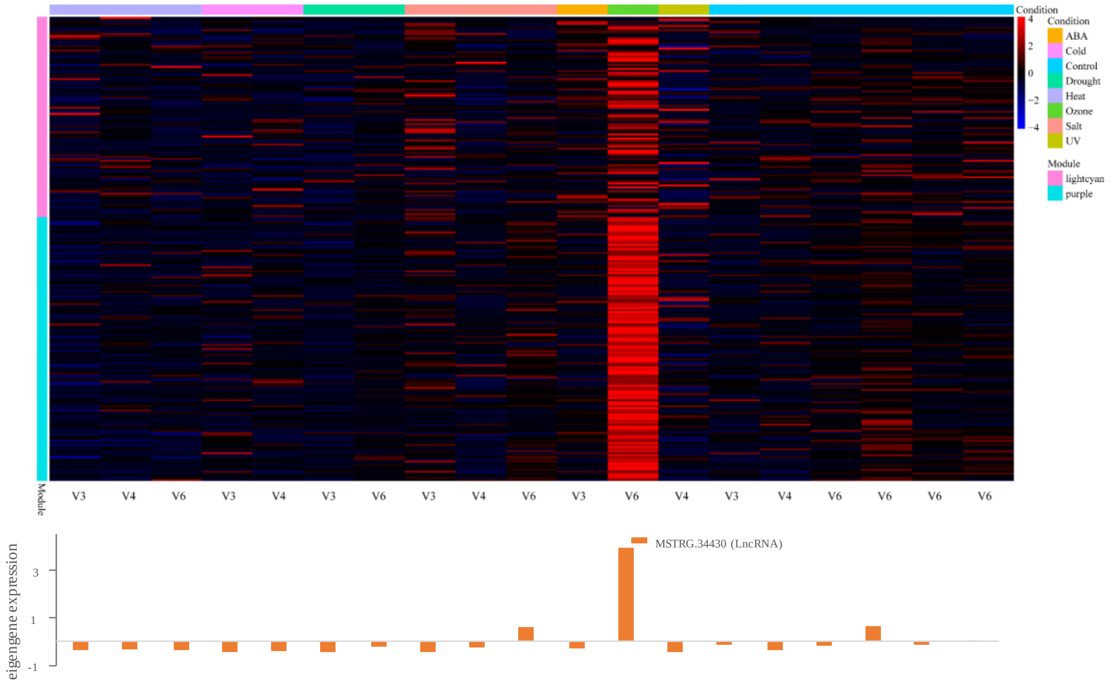

Supplement: Supplementary file 1 — Additional file 1: Figure S1. A schematic showing the data used in this paper, the bioinformatic pipeline for lncRNA identification, numbers of genes and identified lcRNAs, and some features of downstream lncRNA analyses. Figure S2. The chromosomal distribution of lncRNAs. Density was plotted across each chromosome. Figure S3. Differential expressed genes (DEGs) under different abiotic stresses. A-D, Venn diagram of DEGs under different stresses across different development stage V3-V6. E-G, Venn diagram of DEGs under different stress conditions at one stage. Figure S4. The correlation values (r) between each of the inferred coexpression modules and the specific traits (e.g., Heat, Cold) at different development times (e.g., V3 to V6). A subset of these data for the top 16 stress-associated modules is provided as a heat map in Figure 3. Figures S5-S9. These figures are the same format as that of Figure 4, but represent cold (Figure S5), drought (Figure S6), salt (Figure S7), UV (Figure S8) and ozone stress (Figure S9). Each figure contains a heat map (top) and graphs of the expression of specific TElncRNAs (bottom) that were chosen because they overrepresented with high interconnectivity. The heat graph shows transcript expression levels for genes and lncRNAs in each module (y-axis) and across conditions (x-axis). The key to modules (yaxis) and stress conditions (x-axis) are shown on the right legend, with conditions also separated by developmental stage (bottom of x-axis). Warmer colors within the heat map indicate high expression, and cooler colors are under-expression. The bar plots below the heat graph are eigen-lncRNA expression values selected from the top overrepresented lncRNAs with high interconnectivity. The x-axis is the same as the heat map, and the id of the lncRNAs is provided by the color key. [file 12864_2019_6245_MOESM1_ESM.pdf]
